# Role of Surgeon Experience in Pterygium Surgical Outcomes: A Comparative Study Between Ophthalmology Resident and Consultant

**DOI:** 10.7759/cureus.11711

**Published:** 2020-11-25

**Authors:** Bijnya B Panda, Jyotsna Sharma

**Affiliations:** 1 Ophthalmology, Sriram Chandra Bhanj (SCB) Medical College & Hospital, Cuttack, IND

**Keywords:** pterygium excision, conjunctival autograft, surgeon experience, learning curve

## Abstract

Purpose

Although surgical technique and patient variables are responsible for the recurrence of pterygium, the surgeon's experience has been sparsely studied. This retrospective study was designed to compare the surgical time, complications, and rates of recurrence after primary pterygium excision between consultant ophthalmologists and trainee residents.

Material and methods

In this retrospective study, we collected the data of 176 primary pterygium eyes, who were operated on with excision and sutureless/glueless conjunctival autograft either by the consultant (group A) or by the trainee (group B). The demographic profile, surgical time, complications, and recurrences between both groups were analyzed.

Results

Both the groups were comparable with regards to age, gender, religion, side of the eye, size of the pterygium, and duration of follow-up. The mean operative time was longer in group B (26.5+/-3.8 minutes) than group A (14.2+/-1.6 minutes). Though a relatively higher percentage of complications was observed in group B (12% vs. 9%), the difference was statistically not significant (Mann-Whitney U test, p-value 0.271). There was no statically significant difference in recurrence rate (6.8% vs 9.4%) between the groups.

Conclusion

With regards to the role of surgeon experience in primary pterygium excision using the sutureless and glueless conjunctival autografting technique, the residents did not have any statistically significant differences in their postoperative complications and recurrence rates. However, the surgical time was significantly higher in the resident group owing to the learning curve.

## Introduction

Pterygium is a wing-shaped fibrovascular structure of conjunctiva that encroaches onto the cornea [1]. This condition is widespread, but it is commonly associated with increasing age, male gender, rural population, and ultraviolet (UV) light exposure. The pathogenesis of pterygium is still controversial and, currently, the ultraviolet light-induced damage to the limbal stem cell barrier with subsequent conjunctivalization of the cornea has been the most accepted concept [2].

Although surgical treatment is recommended for the definite treatment of pterygium, there is no consensus on the type of surgical procedure [3]. The type of surgical procedure is one of the major variables determining the recurrence in pterygium surgery. The conjunctival autograft (CAG) technique has been widely used as the most successful procedure. Still, alternative methods like inferior autograft, conjunctival rotation autograft surgery, and amniotic membrane transplant have their importance in specific situations [4-5]. All these techniques involve the use of sutures or fibrin glue. Therefore, there is a substantial risk of complications such as infection, granuloma formation, and chronic inflammation. The plasma-derived fibrin glue has the potential risk of prion disease transmission and anaphylaxis in predisposed individuals [6-7]. Mitomycin-C and beta-irradiation have proven to be useful adjunctive therapies in reducing recurrence. However, these are associated with severe complications like limbal stem cell deficiency, scleral necrosis, and the dreaded complication of endophthalmitis [8-9]. The rates of recurrence following pterygium surgery with CAG vary from 5.5%-39 % according to the earlier published studies [10-14].

The young age, fleshy, non-translucent pterygium, and history of a previous recurrence are patient-dependent factors that predispose an individual to recurrence [10-16]. Apart from these patient factors and surgical procedures, the surgeon experience has been sparsely evaluated for pterygium recurrence [17-19]. The study by Farrah and Lee reported an increased incidence of recurrence and complication rates in the trainee group using conjunctival autograft. Similarly, another study by Ti et al. observed 5%-82% recurrence rates with the conjunctival autograft. This wide variation in the recurrence rate was related to a significant learning curve or different surgical techniques for this procedure. While the most experienced surgeon reported a 5% recurrence rate, the inexperienced surgeon had 82% recurrence.

Surgical training is an integral part of the residency program at our institute. We ensure that the resident should be comfortable with pterygium excision with CAG under the supervision of a consultant during the residency. This study was designed to compare the surgical time, complications, and recurrence rates of primary pterygia excision between ophthalmology consultants and trainee residents using the sutureless and glueless conjunctival autograft technique.

## Materials and methods

Study design and patient recruitment

This retrospective study was designed to compare the outcome and complications of pterygium surgery performed by consultants and trainee residents. All patients operated between January 2017 and December 2019 with primary pterygium excision and limbal-conjunctival autograft with at least one-year follow-up were included. The patients who were treated for recurrent pterygium or having dry eye syndrome, collagen vascular diseases, pseudopterygium, or associated conditions, such as glaucoma, diabetes mellitus, Stevens-Johnson syndrome, ocular cicatricial pemphigoid, were excluded. The details of the patients, surgical details, and postoperative complications were collected from the medical records.

Two-hundred forty patients underwent pterygium surgery either by the consultants (group A) or by the trainee residents (group B) during this study period. The consultant ophthalmologist was defined as a surgeon who had a minimum of six years of independent surgical experience, and a trainee resident was defined as a resident who was in his/her last six months of the training period and had performed a minimum of 10 pterygium surgeries. Sixty-four patients (26.6%) who did not meet the inclusion criteria of the required follow-up of one year were excluded. Therefore, 176 patients (176 eyes) were enrolled in the study. The study adhered to the tenets of the Declaration of Helsinki.

Surgical details

All patients were operated on under peribulbar anesthesia with the same surgical technique. The conjunctival autograft (thin and tenon free, 1 mm larger than the host bed) was harvested from the superior conjunctiva and placed in proper limbal orientation after completely drying the host bed. No suture or fibrin glue was used in any of the patients. A waiting time of 5 minutes after the placement of the graft was strictly followed for each surgery. The postoperative regime was the same for both groups and consisted of moxifloxacin (0.5%) and dexamethasone (0.1%) four times a day for a mean duration of four weeks in tapering dosage.

Data collection and follow-up

The demographic details, size of the pterygium, surgical time (excluding peribulbar block and preparation time), and follow-up outcome or complications were entered into a predesigned proforma. Major complications included graft retraction, symblepharon, Dellen, graft loss, necrosis, scleral melt, and corneal scar. Minor complications included intra-epithelial cyst and irregular astigmatism. Graft edema and sub-conjunctival hemorrhages were not considered as minor complications; instead, these features were considered part of the healing process. Early recurrence was defined as the visible fibrovascular tissue near the limbus or extending onto the cornea within six weeks of surgery, and late recurrence as a similar appearance after six months of operation. Outcomes were recorded for each follow-up visit and then combined into totals for the entire follow-up period for each patient. Pterygium was graded depending on the extent of corneal involvement: Grade I - crossing the limbus, Grade II - mid-way between the limbus and pupil, Grade III - reaching up to the pupillary margin, and Grade IV - crossing the pupillary margin. The patients were reviewed on the first postoperative day, at the end of one week, four to six weeks after surgery, and after that, every three to six months.

Statistical analysis

The data were entered into a Microsoft Excel sheet (Microsoft Corporation, Redmont, WA) for subsequent cleansing and statistical analysis. The continuous data were expressed as mean + SD based on the normality of the data. The Student's t-test or Mann-Whitney U test was used to compare the difference between the two groups for continuous parametric or non-parametric data, respectively. The chi-square or Fisher exact test was used to compare the categorical variables. Multiple linear regressions were used to determine the effect of various variables on the outcome. In all cases, the level of statistical significance was kept at 0.05.

## Results

There were 80 patients (male 54 and female 26) operated by the consultant ophthalmologists (group A), and 96 patients (male 62 and female 34) operated by the trainee ophthalmologists (group B). The mean age of the patients in group A and B were 50.1 years (range 27 to 75 years) and 50.9 years (range 27 to 73 years), respectively. The mean length of the pterygia in group A was 3.5 mm (range 2.5 to 4.7 mm) whereas the mean length was 3.4 mm (range 2.0 to 4.8 mm) in group B. There were no signiﬁcant differences between groups A and B with respect to age, gender, religion, side of the eye treated, and size of the pterygium (Table 1).

**Table 1:** Summarizing patient demographics (a=chi-square test, b=Mann-Whitney two-tailed hypotheses,c=Fisher exact test)

Variable	Consultant Cases (Group-A, n=80)		Trainee Cases (Group-B, n=96)	Remarks
Gender	Male	54 (67.5%)	62 (64.6%)	p-value0.684^a^
	Female	26 (32.5%)	34 (35.4%)	
Eye treated	52% Left eyes(42/80)		49% Left eyes(47/96)	p-value 0.639^a^
Average age	50.1+/-11.3 years(27-75)		50.9+/-11.7 years(27-73)	p-value 0.638^b^
Religion	Hindu 45, Muslim 35		Hindu 59, Muslim 37	p-value 0.539^c^
Size of pterygium	3.5+/-0.6 mm		3.4+/-0.7 mm	p-value 0.101^b^

The surgical time in group A was 14.2+/-1.6 minutes, and in group B, it was 26.5+/-3.8 minutes (independent two-paired T-test, p-value < 0.001), i.e, the results are statistically significant.

The mean follow-up period in group A was 614 +/- 226.9 days (range 365 days to1092 days), and in group B, it was 652 +/- 218.5 days (range 365 days to 1104 days). There was no significant difference between the groups with regard to follow-up duration (p-value0.138, Mann-Whitney test).

**Figure 1:**
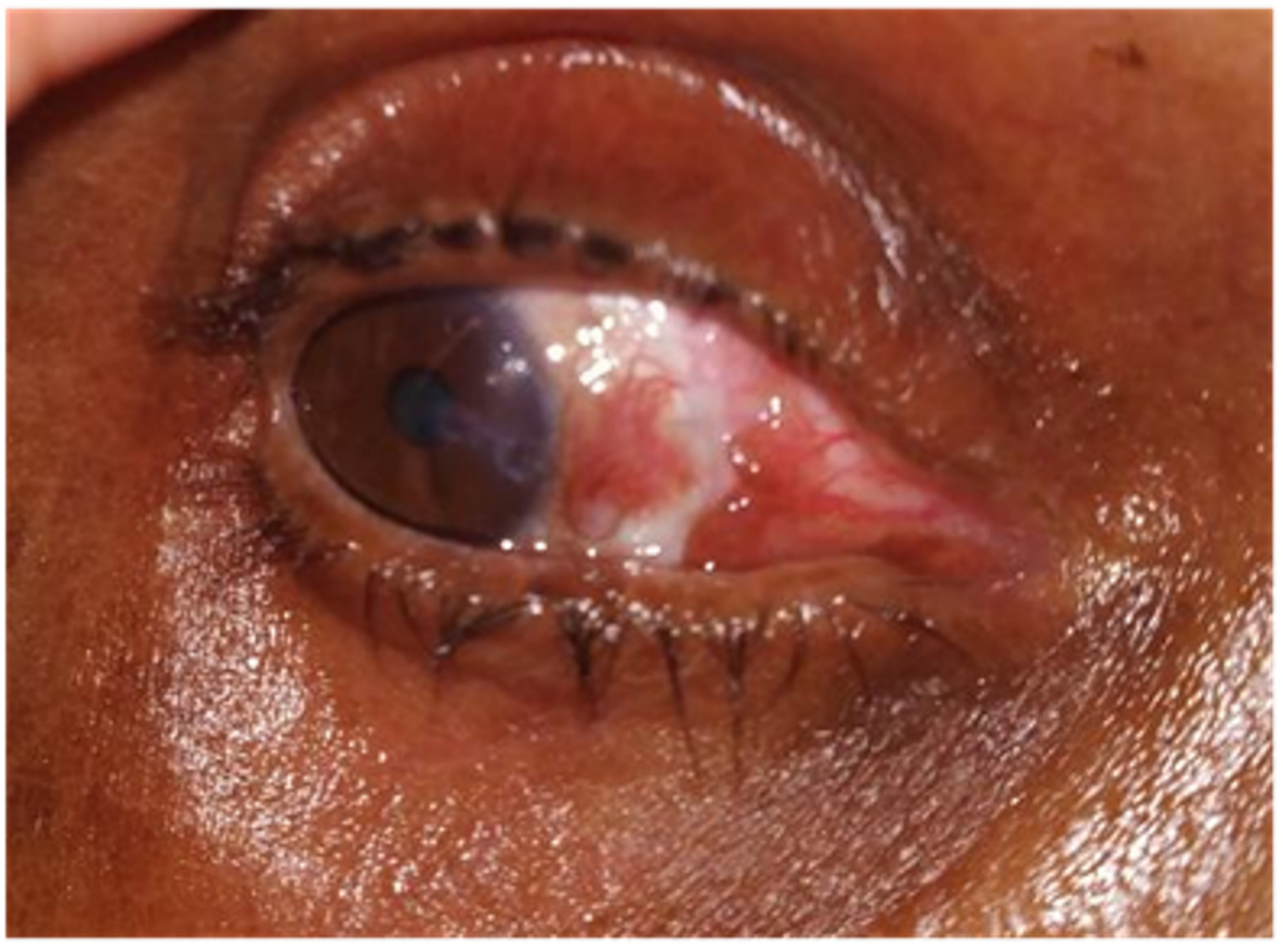
Graft retraction after two months of pterygium excision

**Figure 2:**
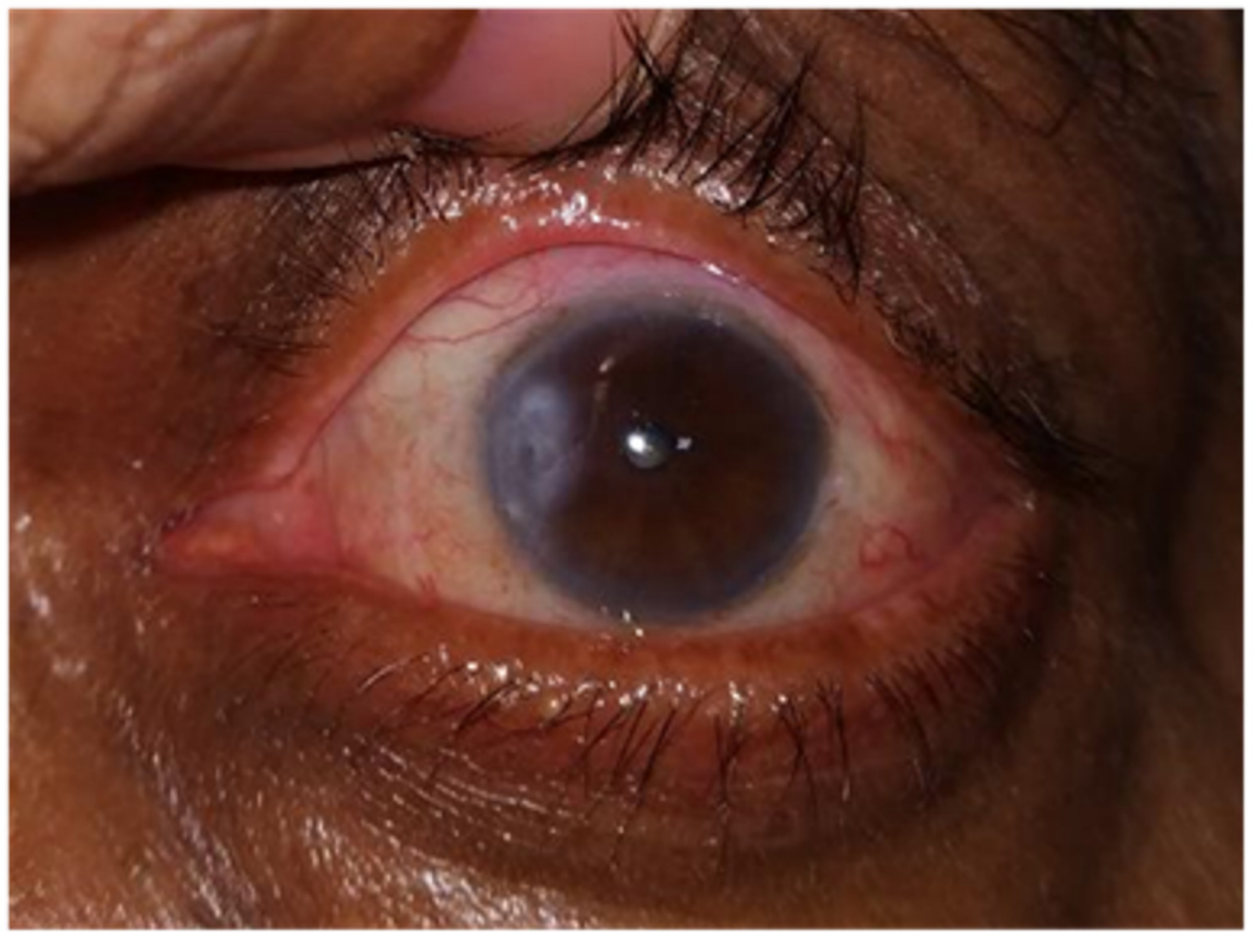
Corneal scar after pterygium excision; CAG in place CAG: conjunctival autograft

There were five eyes with complications in Group A, including two minor (epithelial cyst) and three major complications (graft retraction, and graft loss). Similarly, 12 eyes in group B showed complications, including four eyes with minor complications like intraepithelial cyst formation or irregular astigmatism, and eight eyes with major complications like graft retraction (Figure 1), graft loss, and corneal scarring (Figure 2). The detailed analysis of the individual complications has been shown in Table 2. There were no statistically significant differences in complication rates between both the groups (Mann-Whitney U test, p-values >0.271).

**Table 2:** Summary of the complication rates between the two groups

COMPLICATION	GROUP-A (n=80)	GROUP-B (n=96)
Number of patients with any complication	7%(n=5)	12%(n=12)
Patients with minor complications	3%(n=2)	4%(n=4)
Patients with major complications	4%(n=3)	8%(n=8)
Graft retraction	3%(n=2)	3%(n=3)
Symblepharon	-	-
Dellen	-	-
Graft loss	1%(n=1)	3%(n=3)
Necrosis	-	-
Scleral melt	-	-
Corneal scar	-	2%(n=2)
Cyst	3% (n=2)	3%(n=3)
Irregular astigmatism	-	2%(n=1)

Concerning the recurrence rate, five out of 80 patients (6.8%) in group A showed pterygium recurrence as compared with nine out of 96 patients (9.4%) in group B at the latest follow-up. All eyes showed recurrence after six months of surgery. The recurrence rate in both groups was statistically not significant. (Fisher's exact test, p=0.579).

## Discussion

The majority of the studies that have analyzed the surgical outcomes for pterygia have considered either the patient-related factors, the surgical technique involved, or the adjuvant therapies to explain variables that inﬂuence rates of complications or recurrence [14-16]. But, very few studies have incorporated the surgeon experience as a determining factor in the outcome of pterygium treatment [17-19]. Our study didn't find a statistically significant difference between the consultants and the trainees in the incidence of complications and recurrence rate following primary pterygium excision using the sutureless and glueless conjunctival autograft technique. However, the surgical time was significantly higher in the resident group due to the learning curve.

Apart from cataract surgery, pterygium excision is the most common surgical procedure performed in ophthalmology [3]. Hence, adequate training of the residents to precisely perform the procedure is vital. At the same time, there should be a consideration to minimize the cost and maximize the outcome, as most of the patients are from a rural background, with inadequate resources [3]. Both factors were adequately addressed in this study. The residents were adequately exposed to the procedure during their residency. Even before performing the procedure independently, they operated under supervision. The residents considered in this study had already performed at least 10 procedures beforehand, and they were in their last six months of training. The study by Ti and his associates reported a recurrence of 5% in the hand of the most experienced surgeon who has performed at least 10 surgeries before [18]. They observed high recurrence rates, ranging from 20% to 80% among the surgeons who had never performed the procedure before or had performed only one procedure. Farrah et al. also reported a recurrence of 19.4% in the trainee group and 6.8% in the trained consultant group [17]. Their findings suggest that the experience of the surgeon can influence success rates and complications. However, the residents of varying years of training (first year to the third year) were included in their study and there was no mention of their previous surgical expertise. There is no point in comparing an inexperienced or first-time surgeon with an experienced surgeon and demonstrating a superior outcome in the latter group. Janson and Sikder compared the attendee and the residents for different surgical techniques for pterygium excision [19]. They reported a significant difference between the groups for the conjunctival autograft surgery technique, but this difference was not observed when concomitant mitomycin C was used. Eighty-one percent of residents used mitomycin C in the conjunctival autograft, whereas only 38% of attendees used it. Based on the previous study and our observation, it was apparent that surgeons with at least 10 independent surgical procedures had a good outcome [18]. The teaching faculties should encourage the students to acquire the efficiency of performing this easy and efficient technique for primary pterygium excision while taking care of certain principles and utmost precautions during the surgery.

It has been proven that the conjunctival autografting technique is the most superior procedure in pterygium surgery [3]. Nevertheless, other alternative techniques and adjuvant therapies have their own importance. But, in developing countries where the paying capacity of the patients is less, this simple yet efficient procedure of sutureless and glueless conjunctival autograft procedure could cut short the cost of the burden involved [3]. The surgical outcomes of this procedure in terms of cosmesis and recurrence have also been proven to be superior to other procedures [18]. There are other procedures that utilize amniotic grafts in specific cases, such as scarred conjunctiva as in trachoma, or in patients who require glaucoma filtration surgeries [19-20]. However, many clinical trials and meta-analyses prove that amniotic membrane grafts have more chances of recurrence than conjunctival autograft [21-22]. We did not use the amniotic grafts and fibrin glue in our study as they are costlier and require logistics which were difficult to procure in our set-up. We did not include suture or mitomycin-C assisted techniques in our study because of cost and limited availability. We did not perform primary conjunctival closure or the bare sclera technique, as those techniques are considered inferior and have fallen out of favor for even general ophthalmologists [23]. In our study, which is the first of its kind, we have been able to demonstrate that sutureless and glueless conjunctival autografting is equally efficient in both consultant and resident groups.

There was an important observation in this study. The surgical time was significantly higher in the trainee group, and it was almost double the time needed by an experienced consultant. This can be attributed to the learning curve of the residents and the time required in obtaining a thin and tenon free graft. The principles of dissection of the pterygium from its bed were the same in both the groups. We believe in minimally invasive conjunctival excision of around 2 mm from the limbus followed by avulsion of the head with the help of a Lim's corneoscleral forceps and then clearing the remnants off from the cornea with a crescent blade. The importance of excising the underlying diseased tenon tissue just below the body of the pterygium rather than excising the conjunctiva cannot be underestimated. The waiting time for graft adherence was the same in both the groups, and hence this was not a bias to interpret the surgical duration. It is also well-known that the more the surgical time, the more the handling of tissues and resulting inflammation postoperatively, which may be a reason behind the recurrence of pterygium. the dissection of the pterygia. Although a slightly higher incidence of recurrence was observed in the trainee group, it was not statistically significant.

A recent study by Ghoz et al. in 2018 found that all patients undergoing conjunctival autograft will have graft edema due to transudation from blood vessels and patchy subconjunctival hemorrhage in the first week; hence, these changes should not be considered as a complication rather a part of reperfusion injury process [24]. Taking this fact into consideration, we have not included graft edema and subconjunctival hemorrhages in the list of complications in our study. When we analyzed complication rates in both groups, we could infer that there existed a few differences, but the difference was statistically not significant. We observed a greater percentage of cases with graft retraction, graft loss, and corneal scarring in the resident cases, which could be attributed to the lack of experience in a trainee surgeon or a difference in the postoperative medication compliance in those cases. A reporting bias could also be the reason where the surgeon reports higher complication rates in the residents' cases. Farrah et al. reported a higher complication in the resident group, as they had difficulty in obtaining an adequately sized, tenon-free graft, which they named as an activated tenon [17]. They also mentioned that the resident performs a vigorous surgery (a surgical trauma) technique rather than the gentle handling of tissues [17].

The recurrence after pterygium is multifactorial, and it is usually observed within the first 12 months of surgery. Hirst et al. evaluated 161 recurrent pterygia and found that there was a 50% chance of recurrence within the first 120 days, and 97% of recurrences were observed within a year [25]. The minimum follow-up time of one year in this study is hence justified. We believe the reason for relatively higher recurrence rates among the residents in our study may be attributed to three factors; firstly, the technique of obtaining the graft needs expertise to adequately clear the subconjunctival tissue along with the excision of the pterygium. Such clearance may even require careful hooking of the rectus muscle. Secondly, the excessive use of cautery by novice surgeons may result in more necrotic tissue and consequent poor healing and early recurrence. Moreover, surgical time taken by the residents is comparatively higher, which may result in more postoperative inflammation and, consequently, recurrence. Campagna et al., in 2018, retrospectively evaluated the recurrence rates after primary pterygium excision by trainee surgeons in patients of different races and surgical techniques [18]. They concluded that the level of surgeon experience was not related to the recurrence rates; rather, the racial difference (more in the Hispanics and black races) was more important. However, we did not observe any difference in recurrence rate between the Hindu and Muslim populations of the Indian subcontinent. 

There are a few limitations to this study. The major limitation was the retrospective study design, as the data were generated based on the medical records narration. Advanced techniques, such as the use of amniotic grafts or suture/glue-assisted surgeries, were not considered for the comparison. However, this study had an adequate number of patients and the minimum time of one-year follow-up was sufficient to determine the recurrence of the pterygium. The major strength of this study is that one particular surgical technique has been compared between the consultants and trainees where the trainee has obtained a certain level of surgical experience to precisely execute the procedure. 

To conclude, there were no significant differences in recurrence rate and complications between the consultants and trainee residents in pterygium excision surgery using a conjunctival autograft technique. The time taken for the procedure was considerably higher among the residents. Further prospective randomized controlled studies are needed to better establish the role of surgeon experience in pterygium surgery performed using different surgical techniques. 

## Conclusions

To conclude, there were no significant differences in recurrence rate and complications between consultants and trainee residents in pterygium excision surgery using a sutureless and glueless conjunctival autograft technique. The time taken for the procedure was considerably higher among the residents. Further prospective randomized controlled studies are needed to better establish the role of surgeon experience in pterygium surgery performed using different surgical techniques.
